# Effects of different temperatures on *Leiocassis longirostris* gill structure and intestinal microbial composition

**DOI:** 10.1038/s41598-024-57731-6

**Published:** 2024-03-26

**Authors:** Zhongmeng Zhao, Han Zhao, Xiongyan Wang, Lu Zhang, Chengyan Mou, Zhipeng Huang, Hongyu Ke, Yuanliang Duan, Jian Zhou, Qiang Li

**Affiliations:** 1 Fisheries Institute, Sichuan Academy of Agricultural Sciences, Chengdu, Sichuan China; 2 Sichuan Water Conservancy Vocational College, Chongzhou, Sichuan China; 3 Present Address: 1611 Xiyuan Avenue, Chengdu, China

**Keywords:** Microbiology, Microbial communities

## Abstract

Fish are poikilothermic vertebrates and their physiological activities are affected by water temperature. In recent years, extreme weather has occurred frequently, and temperature changes have adversely affected the growth of farmed fish. To explore the changes in gill tissue structure caused by changing the water temperature and the relationship between the intestinal microbiota and the *Leiocassis longirostris* host adaptation mechanism, gill tissue sections and intestinal microbial 16S rRNA amplicon sequencing were conducted under different temperature stress (low temperature 4 °C, normal temperature 26 °C and high temperature 32 °C). The results showed that heat stress and cold stress caused injury and swelling, terminal congestion, cell vacuolation, and necrosis of the gill tissue of *L. longirostris*. For intestinal microbiota, the abundances of Pseudomonadota and Bacillota increased at the cold stress, while the abundances of Fusobacteriota and Bacteroidota increased at the heat stress. The number of opportunistic bacteria, mainly *Aeromonas* and *Acinetobacter*, was the highest under cold stress. In addition, the richness of the intestinal microbiota decreased significantly at heat and cold stresses, while evenness increased. Prediction of intestinal microbiota function showed that most common functions, such as metabolism of cofactors and vitamins, energy metabolism and replication and repair, were decreased significantly at heat stress and cold stress, and phylogenetic relationship analysis revealed significant differences among the groups. In conclusion, the change of temperature altered the gill tissue structure, and affected the structure and homeostasis of the intestinal microbiota, thus affecting the survival time of *L. longirostris*, and cold stress had a greater effect than heat stress.

## Introduction

*Leiocassis longirostris* a freshwater fish in the family Bagridae. It is widely distributed in the Yangtze, Liao, and Huaihe Rivers of China. *L. longirostris* is a sought-after commercially important species^1^. However, overfishing, environmental pollution, construction of water conservancy projects, and habitat degradation and fragmentation of wild *L. longirostris* populations have contributed to sharp declines. Thus, artificial culture was initiated to alleviate the increasing demand for *L. longirostris*^2^.

The gastrointestinal tract is an important immune organ in fish and plays a very important role in the resistance to pathogens. This important function of the gut is largely the responsibility of the intestinal microbiota. Intestinal microorganisms form a large microbial population, and according to the state of the host organism, they are always in a dynamic change and mutually-beneficial symbiosis with the host^3^. Research on the function of the intestinal microbiota has become increasingly in-depth and specific. As an “extra organ” of fish, the intestinal microbiota directly or indirectly assists the body in various physiological and metabolic activities^4^. One of the most important functions is the metabolism of substances^5^. In addition, intestinal microbiota act as a natural biological barrier for the host. The microbial flora covers the surface of the intestinal mucosa, effectively reducing damage to intestinal epithelial cells caused by sudden external stimulation. When pathogenic bacteria enter the intestine with food, the intestinal microbiota is the first barrier encountered. Some microorganisms directly eliminate some pathogens, and the intestinal microbiota stimulates the intestinal mucosa to secrete bactericidal substances to maintain normal immune function^6^.

The differences in the composition of fish intestinal microbiota communities are caused by differences in feeding habits among fish^7–9^. However, changes in environmental temperature also have a significant impact on the composition of fish intestinal microbiota. Environmental temperature is an important abiotic variable, which affects the adaptation of the fish to the environment^10^. Changes in ambient temperature affect the abundance of the host intestinal microbiota and the concentrations of metabolites^11^. The number of intestinal microbiotas in red hybrid tilapia at 18 °C was higher than that at 26 °C^12^. The study results of Yoshimizu et al. showed that the community structure of the intestinal microbiota of fish varied to a certain extent in different seasons. The number of intestinal microbiotas was the lowest in winter and the highest in summer^13^. In addition, ambient temperature induces changes in the intestinal microbiota and affects host adaptation, including resistance to intestinal colonization, host energy and nutrient assimilation, and host immunity^10^. Animals living under extreme temperature conditions may have specific microbiota in their intestine, and these temperature-induced microbiota play an integral role in maintaining host health, growth, and development^14^.

The histological changes in the gills are widely used as a health status indicator in fish. The large surface area of the gills is in contact with the water, and histological and subcellular changes effectively reflect the degree of body injury^15^. Heat stress causes gill damage in *Salmo salar* and *Paralichthys olivaceus*, such as swelling of the lamella, high peeling, and rupturing of the epithelium^16,17^. Under the stress of high temperature and low temperature in *Oryzias latipes*, the gill morphology would change, different degrees of oxidative stress would be generated, and a large number of ROS would be released, thus inducing the apoptosis of gill tissue^18^. Although *L. longirostris* has a wide temperature adaptability range, it often experiences temperature stress during production. Therefore, studying the histological changes in gills and intestinal microbiota of *L. longirostris* under various temperature stress levels is significant in guiding production, and can provide a theoretical basis and preventive measures for production and breeding practices.

## Materials and methods

### Fish of the study

The experimental animals were *L. longirostris* bred by the Fisheries Research Institute of Sichuan Academy of Agricultural Sciences. *L. longirostris* at 3 months of age, similar body length and weight (body length, 10.45 ± 0.26 cm; body weight, 18.32 ± 1.11 g), normal swimming behavior and no physical injury was selected as the experimental fish. Before the formal experiment, *L. longirostris* were acclimated for 2 weeks with water temperature (26 ± 0.5) °C, dissolved oxygen ≥ 5 mg·L^−1^, 24 h aeration, 14 h light/10 h dark cycle, and fed twice a day (9:00 am and 5:00 pm). The daily feeding amount is 3% of the body mass of the fish, and the water is changed every 3 days.

### Temperature stress experiment

**Figure 1:**
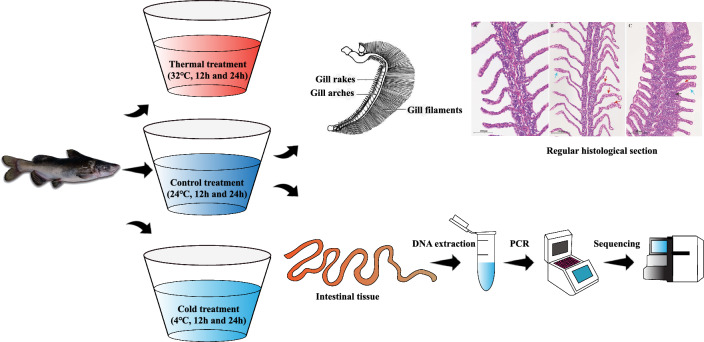
Experimental procedure. *L. longirostris* was placed in the water temperature of 32 °C, 26 °C and 4 °C respectively, and its gills and intestines were taken at 12 h and 24 h for the experiment.

*L. longirostris* can survive at 0–38 °C, but the optimum growth temperature is 25–28 °C^19^. Therefore, the experiment consisted of 3 groups, cold stress group (4 °C), heat stress group (32 °C) and normal temperature (26 °C) control group. In the control group, the cold and heat stress groups were subjected to gradient cooling or heating at a rate of 1 °C/h starting from 26 °C. Each group comprised of six replicates with 10 fish in each, and placed in incubators with constant temperature. Samples were collected after 12 h and 24 h of stress. In this experiment, H12, H24, C12, C24 and CK represent 12 and 24 h at 32 °C, 12 and 24 h at 4 °C and 24 h at 26 °C, respectively (Fig. 1).

### Section of gill tissue

One *L. longirostris* was selected from each parallel of the three groups after 24 h of stress. After anesthetizing the fish in MS-222 solution, the gill tissue was dissected immediately and fixed overnight in a 4% volume fraction polyformaldehyde solution. After dehydration with gradient ethanol, the gill tissue was treated with xylene for 1 min and then immersed in wax for 3 h. The gill tissue was sliced with a Leica microtome at a thickness of 5 μm, and the slices were maintained overnight in a 37 °C dryer. The sections were taken for hematoxylin–eosin (H&E) staining^20^. Three stained gill plates were used from each group for analysis. At least five fields of view were observed for each section under an optical microscope (TS-200B, SDPTOP, Ningbo, China), and the most representative field of view was selected as the final result (Fig. 1).

### Gut microbiome

After 12 h and 24 h under the different temperature stressors, six tails (two/parallel) were randomly selected from each group. After anesthesia, the spine was incised and the fish was euthanized in a sterile environment. The intestine was dissected and separated in normal saline. Prior to anatomical examinations, all fish specimens were administered a thorough anesthesia protocol in accordance with local standard practices. This procedure involved immersing the fish in a solution containing 100 mg/L of MS-222 until they exhibited no response upon manual tail manipulation, signifying a state of profound anesthesia. After documenting their weight and length measurements, the fish were euthanized via caudal venipuncture. The outer wall of the intestine was flushed with normal saline three times to remove the intestinal contents. Subsequently, the intestinal contents of two *L. longirostris* from the same aquarium were pooled into 1 sample, and six samples were used as parallel groups. The 16S rRNA amplicon sequencing was completed by Guangzhou Gidio Biotechnology Co., Ltd. (Guangzhou, China). Total genomic DNA was extracted using the FastDNA^®^SPIN Kit for Soil (MP Biomedical, Santa Ana, CA, USA). Genomic DNA integrity was determined by agarose gel electrophoresis. The concentration and purity of the genomic DNA were determined by Nanodrop 2000 and Qubit3.0 spectropheatometers. The 341F (5′-CCTACGGGNGGCWGCAG-3′) and 806R (5′-GGACTACHVGGGTATCTAAT-3′) primers were used to amplify the V3–V4 hypervariable region of the 16S rRNA gene. The sequencing was performed using an Illumina NovaSeq 6000 sequencer (Fig. 1).

### Statistical analysis

To get high quality clean reads, raw reads were further filtered using FASTP^21^ (version 0.18.0). Paired end clean reads were merged as raw tags using FLASH^22^ (version 1.2.11) with a minimum overlap of 10 bp and mismatch error rates of 2%. Noisy sequences of raw tags were filtered under specific filtering conditions^23^ to obtain the high-quality clean tags. The clean tags were clustered into operational taxonomic units (OTUs) of ≥ 97% similarity using and UPARSE^24^ (version 9.2.64) pipeline. All chimeric tags were removed using and UCHIME algorithm^10^ and finally obtained effective tags for further analysis. The representative OTU sequences were classified into organisms by a naive Bayesian model using RDP classifier^25^ (version 2.2) based on SILVA database^26^ (version 138.1), with the confidence threshold value of 0.8.

The community composition stacked bar plot was visualized in the R project ggplot2 package (version 2.2.1)^27^. Biomarker features in each group were screened using LEfSe software (version 1.0)^28^, the randomforest package (version 4.6.12)^29^, the pROC package (version 1.10.0)^30^, and the labdsv package (version 2.0-1) in R^31^. The OTU rarefaction and rank abundance curves were plotted in the R ggplot2 package (version 2.2.1)^27^. The alpha index comparison between the groups was calculated using Welch’s *t*-test and the Wilcoxon rank test in the R vegan package (version 2.5.3)^32^. Sequence alignment was performed using Muscle (version 3.8.31) and the phylogenetic tree was constructed using FastTree (version 2.1). The weighted and unweighted unifrac distance matrix was generated with the GuniFrac package (version 1.0) in R. Sequence alignment was performed using Muscle (version 3.8.31) and the phylogenetic tree was constructed using FastTree (version 2.1). The weighted and unweighted unifrac distance matrix was generated using the GuniFrac package (version 1.0) in R^33–35^. The KEGG pathway analysis of the OTUs was inferred using PICRUSt (version 2.1.4)^36^. The microbiome phenotypes of the bacteria were classified using BugBase^36–38^.

## Results

### Changes in *L. longirostris* gill structure at different temperatures

**Figure 2:**
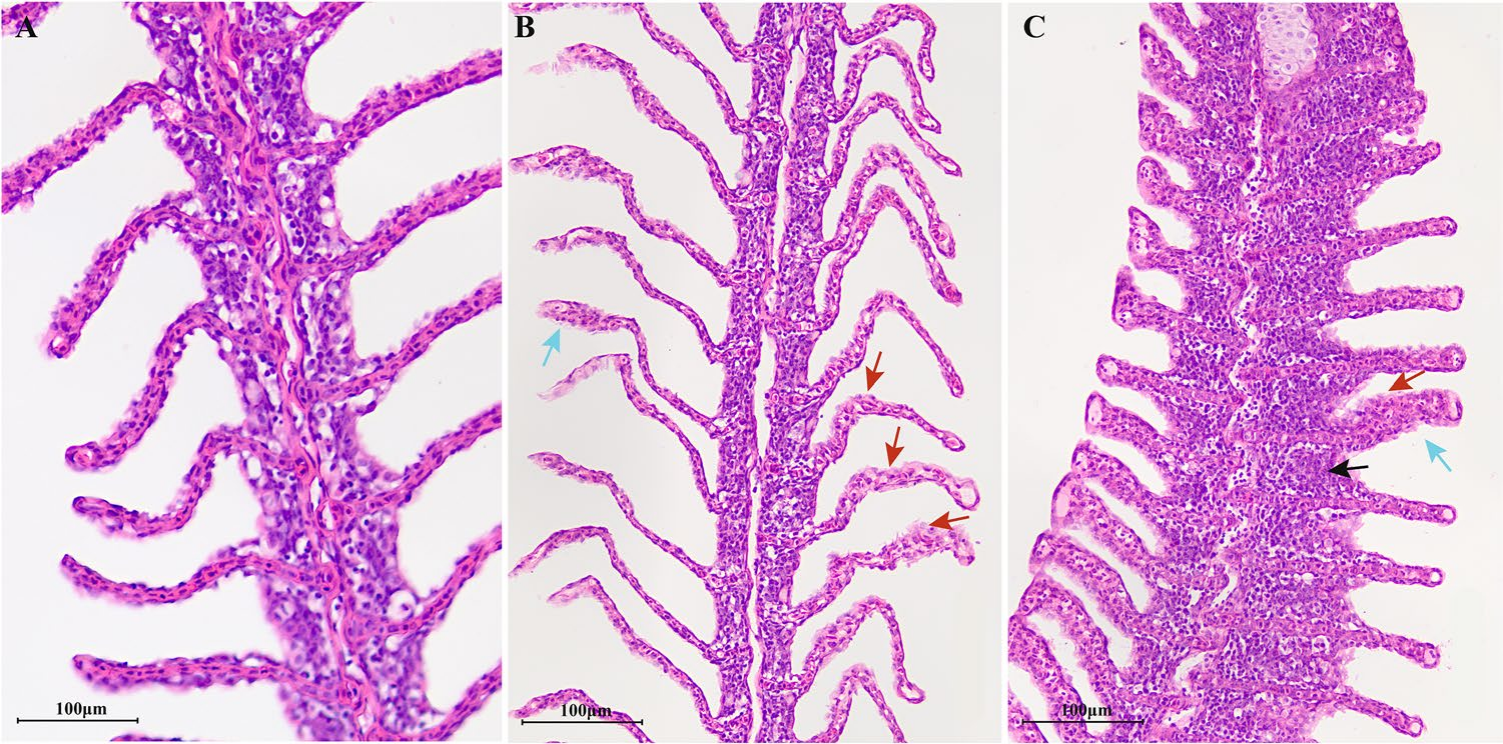
Microstructure of *L. longirostris* gills at different temperatures. (**A**) represent the control group. (**B**) represent the heat stress group. (**C**) represent the cold stress group. Note: Red arrow point to swelling of epithelial cells; blue arrow point to lamellar deformation; black arrow point to basal cell hyperplasia. Scale bar: 100 μm.

*L. longirostris* under cold stress remained at the bottom of the tank, moved slowly, and consumed less food. Under heat stress, *L. longirostris* swam frequently, their gills turned red, food intake decreased significantly, and no deaths were reported. The results of tissue sections showed that the gill filaments from the control fish had a normal structure, uniform color, regular arrangement of the layered structure, and normal morphology of the gill slices and epidermal cells (Fig. 2A). The structure of the gills changes under cold- and heat-stress. Under heat stress, the branchial lamella exhibited extensive edema, with highly exfoliated and ruptured epithelial cells, terminal hyperemia, cell vacuolation and necrosis, and some fusion of the branchial lamellae (Fig. 2B). In addition to swelling of epithelial cells and lamellar deformation, basal cell proliferation also occurred under cold stress (Fig. 2C). These results indicate that both high and low-temperature stressors cause structural changes in *L. longirostris* gills, which may affect survival.

### Sequencing characteristics of the *L. longirostris* intestinal microbiota

**Figure 3:**
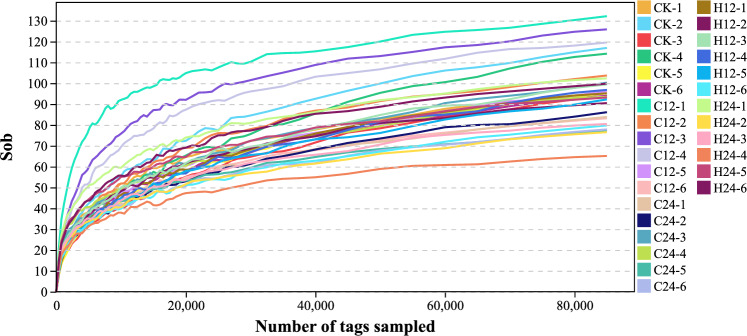
Dilution curves for the Sob index of the 16S rRNA gene MiSeq sequences from the different temperature samples. Different colored lines represent different samples. The horizontal and vertical coordinates respectively represent the number of tags extracted and the number of tags extracted corresponding to the calculation of the diversity index value. *CK* control group, *C12* 12-h cold stress group, *C24* 24-h cold stress group, *H12* 12-h heat stress group, *H24* 24-h heat stress group.

We obtained 3,851,442 raw tags from the 30 fish samples with overlap assembly, and 3,837,327 clean tags after quality control. The remaining 3,543,079 effective tags were obtained after removing the chimeric tags detected in the clustering analogy pairs. The number of effective tags in all samples accounted for more than 85.0% of the number of original raw pair-end reads (Table 1). The rank abundance curves of all samples were obtained based on the OTU abundance of each sample and its ranking. Figure 3 shows the richness and evenness of the species composition in each sample.

**Table 1:** Number of raw tags, clean tags, effective tags, OTUs, and effective ratio (%) for the 16S rRNA libraries in the fish samples.

Samplings	Raw tags	Clean tags	Effective tags	Effective ratio (%)	OTUs
CK-1	130,091	129,286	117,713	88.58	100
CK-2	126,325	125,744	120,194	93.4	126
CK-3	133,184	132,337	126,236	92.97	106
CK-4	120,024	119,539	111,558	91.2	123
CK-5	120,422	119,692	113,961	92.69	102
CK-6	123,106	122,944	112,902	90.09	100
D12-1	134,059	133,701	125,512	91.77	139
D12-2	120,010	119,865	115,684	94.82	109
D12-3	129,646	129,412	124,851	94.3	134
D12-4	133,809	133,571	125,653	92.13	126
D12-5	133,615	133,454	124,705	91.92	104
D12-6	131,549	131,323	115,982	86.79	87
D24-1	121,403	121,192	113,686	92.02	87
D24-2	124,322	124,085	111,496	87.56	88
D24-3	134,539	134,357	122,915	89.85	107
D24-4	134,539	134,367	122,204	89.34	99
D24-5	128,684	128,488	116,806	89.17	80
D24-6	119,176	118,983	108,171	88.98	80
G12-1	131,818	131,119	123,945	92.27	104
G12-2	132,977	132,089	121,802	89.55	97
G12-3	129,043	128,437	118,241	89.97	105
G12-4	126,220	125,862	115,252	89.92	104
G12-5	121,723	120,917	113,576	91.18	98
G12-6	134,810	133,873	124,159	90.13	86
G24-1	133,035	132,422	117,704	86.44	106
G24-2	132,051	131,331	118,244	87.67	81
G24-3	122,057	121,415	112,330	90.23	84
G24-4	133,800	133,049	120,935	88.61	69
G24-5	132,144	131,739	118,890	88.09	98
G24-6	123,261	122,734	107,772	85.8	101

*OTU* operational taxonomical unit, *CK* control group, *D12* 12-h low-temperature treatment, *D24* 24-h low-temperature treatment, *G12* 12-h high-temperature treatment, *G24* 24-h high-temperature treatment.

### Differences in *L. longirostris* intestinal microbiota composition at different temperatures

**Figure 4:**
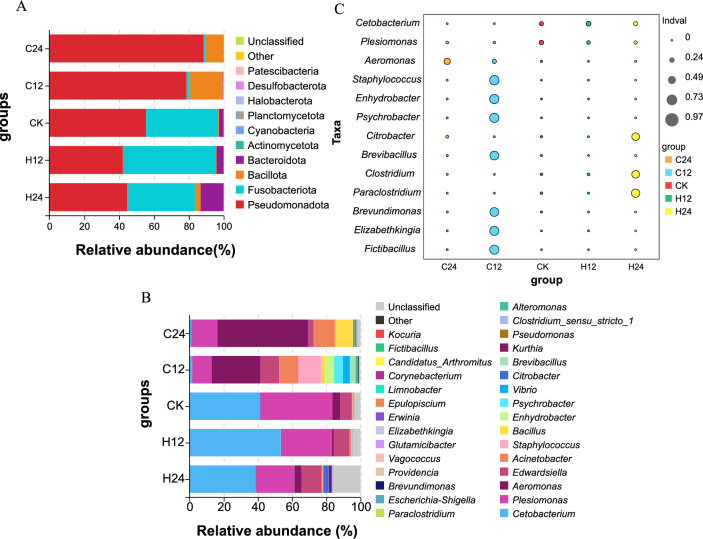
Changes in the intestinal microbiota composition after different temperature stressors. (**A**) Distribution of intestinal bacterial phyla in the experimental treatment and control groups. (**B**) Distribution of intestinal bacteria genera in the treatment and control groups. (**C**) Indicator analysis between the treatment groups. The size of the bubble represents the IndVal value of the species in the corresponding group, and the color of the bubble indicates group information. The larger the value, the more likely the species is an indicator species of that group.

The top 10 dominant bacteria phyla in the intestinal microbiota of the 30 samples accounted for more than 99.93% of all sequence reads. Pseudomonadota dominated the intestinal microbiota from the treatment groups, all of which were more than 42.14%. The contents of Pseudomonadota and Bacillota increased significantly after the cold stress, while Bacteroidota content decreased significantly. In addition, Bacteroidota content increased significantly after the heat stress, while Pseudomonadota content decreased (Fig. 4A). The bacterial composition of the heat-stressed and CK groups was similar based on a bacterial community analysis at the genus level, including *Cetobacterium* and *Plesiomonas*, followed by *Edwardsiella*. *Cetobacterium* and *Plesiomonas* contents decreased significantly in the intestinal microbiota community after cold stress, while *Aeromonas*, *Acinetobacter*, *Bacillus*, and *Psychrobacter* contents increased significantly (Fig. 4B).

According to the index analysis of the abundance and frequency of species in the samples among the cold-stressed groups, the heat-stressed groups and the CK group, the index values of *Aeromonas* in C24, *Staphylococcus* in C12, *Plesiomonas* in CK, *Cetobacterium* in H12 and *Paraclostridium* in H24 were the highest, indicating that these five genera may be C24, C12, CK, H12 and H24 indicator species, respectively (Fig. 4C).

### Diversity analysis of the *L. longirostris* intestinal microbiota under different temperatures

**Figure 5:**
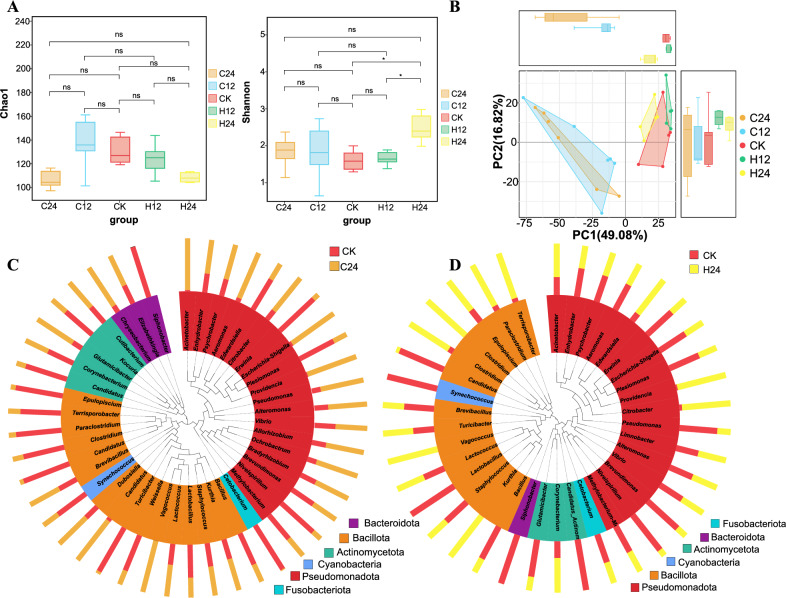
Diversity of bacteria in the gut of the treatment groups. (**A**) Box chart of the inter-group differences in the Chao 1 and Shannon indices. (**B**) Principal Component Analysis (PCA) based on OUT abundance information of species. (**C**,**D**) Phylogenetic relationship among the genera of horizontal species. The phylogenetic tree was constructed with representative sequences of the genera of horizontal species. The colors of the branches and fan-shaped branches represent the corresponding gates, and the stacking histogram outside the fan ring represents the abundance of the genus in the samples.

Alpha diversity refers to the diversity within a particular environment or ecosystem. This index was mainly used to reflect the richness and evenness of species. Compared with the control group, the Chao1 diversity index of the *L. longirostris* intestinal microbiota showed no difference in C12 and H12 groups, but decreased in C24 and H24 groups (Fig. 5A). These results indicate that 24-h heat and cold stress decreased the richness of the *L. longirostris* intestinal microbiota. No significant difference in the Shannon diversity index was observed in the C24, C12 and H12 groups, but it increased significantly in the H24 group, indicating that the evenness of the intestinal microbiota improved significantly under 24-h heat stress (Fig. 5A). These results indicate that 24-h heat and cold stress reduced the richness of the *L. longirostris* intestinal microbiota.

The closer the samples were to each other, the more similar their microbiome structures were. Principal Component Analysis (PCA) based on the OTU abundance information of species found that the samples under different temperature stress were divided into the same group, in which the distance between the heat stress group and the control group was closer, while the distance between the cold stress group and the control group was farther, indicating that the intestinal microbiota of the heat stress group and the control group were more similar (Fig. 5B). In addition, according to the bacterial evolutionary tree analysis of C24 and H24 groups with large changes, the number of bacteria in *Acinetobacter*, *Citrobacter*, *Aeromonas*, *Providencia*, and *Vagococcus* in the C24 group was significantly higher than that in the CK group, while the number of bacteria in *Cetobacterium*, *Erwinia*, and *Turicibacter* was significantly lower than that in the CK group (Fig. 5C). The numbers of *Providencia*, *Citrobacter*, *Vagococcus*, and *Kurthia* were significantly higher in the H24 group than those in the C group, and the numbers of *Synechococcus*, *Lactobacillus*, and *Bacillus* were significantly lower than those in the CK group (Fig. 5D).

### Prediction of intestinal microbiota function of *L. longirostris* under different temperature stressors

**Figure 6:**
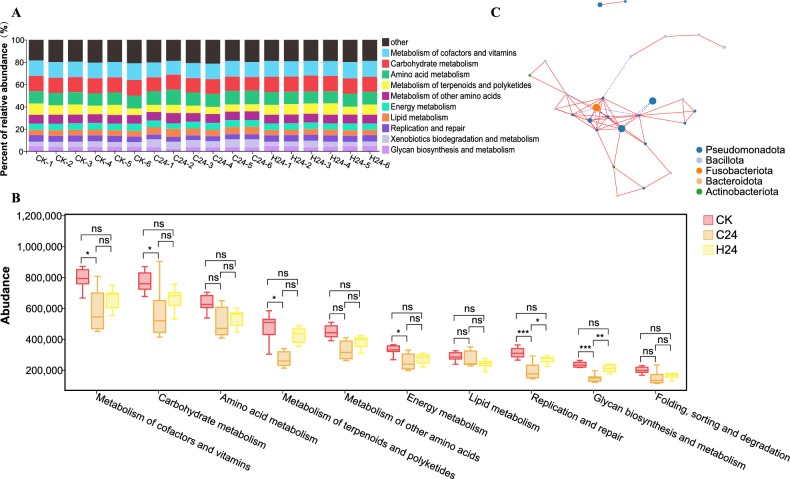
Functional prediction of the intestinal microbiota. (**A**) Histogram of the functional abundance of each sample. The ordinate represents the percentage of relative abundance (%). (**B**) Tukey Honestly Significant Difference (HSD) rank sum test of functional significance difference between different groups. The ten functions predicted to have the highest abundance were compared for differences between the groups, with an asterisk indicating significance. (**C**) Network diagram of the microbial community structure. The circles represent the bacteria, the lines represent the relationships between different bacteria, with red representing positive correlations and blue representing negative correlations.

Based on the PICRUSt functional prediction, the KEGG functional abundance of the sample is obtained. According to the KEGG database functional prediction analysis, all the intestinal microbiota samples in the CK, C24 and H24 groups are mainly involved in the metabolism of cofactors and vitamins, carbohydrate metabolism, amino acid metabolism, metabolism of terpenoids and polyketides, metabolism of other amino acids, energy metabolism and lipid metabolism (Fig. 6A). The KEGG database shows that there were differences in the major metabolic functions between the different treatment groups. Overall, the metabolism of cofactors and vitamins, carbohydrate metabolism, metabolism of terpenoids and polyketides, energy metabolism, replication and repair and glycan biosynthesis and metabolism were significantly lower in the C24 group than in the CK group (*p* < 0.05). The functions of replication and repair and glycan biosynthesis and metabolism under the H24 group were significantly lower than those of the CK group (*p* < 0.05) (Fig. 6B). The analysis of the bacterial interaction showed that Pseudomonadota was the most important bacteria in the *L. longirostris* intestinal microbiota and was related to most of the other bacteria (Fig. 6C).

### Ethical approval

All animal handling procedures were approved by the Animal Care and Use Committee of the Fisheries Research Institute, Sichuan Academy of Agricultural Sciences (Chengdu, China), following the recommendations in the ARRIVE guidelines, under permit number 20210307001–5. At the same time, all methods were carried out by relevant guidelines and regulations.

## Discussion

Too high or too low of a water temperature destroys homeostasis and causes a stress response. A continuous stress state destroys the defense system, eventually leading to the death of the fish^39^. Gills are the most important functional organs of fish, as they are involved in respiration, osmosis, and nitrogen excretion^40^. Cold stress and hypoxia change the gill morphology of *Carassius auratus* by decreasing cell proliferation and inducing apoptosis^41,42^. In addition, a study on gill tissue apoptosis in *Danio rerio* and *Oreochromis niloticus* under cold stress of 8 °C for 12 h reported that apoptosis of the gill was the most obvious among the eight organs in which it was detected, suggesting that the gills are sensitive organs to perceive external changes^43^. In addition to cold stress, heat stress increases peroxide content in mitochondria and intracellular reactive oxygen species (ROS), leading to oxidative damage^44^. The gills of pikeperch are sensitive to heat stress and pikeperch actively responds to heat stress through regulating the antioxidant system, and the expression of Hsp genes and genes involved in energy metabolism^45^. This study observed swelling of gill epithelial cells under the cold- and heat-stress. This change in gill tissue structure can prevent stress factors from penetrating deep tissues and has a protective effect on fish gills. However, it comes at the cost of reduced gas and material exchange^46^. Furthermore, the fusion of gill plates can directly decrease the respiratory surface area, which reduces the impact of stress factors on the gill interior^47^. However, this reduction in respiratory surface area may result in issues such as hypoxia and respiratory failure. The degree of apoptosis of gill cells under cold stress and heat stress and the reasons requires further study.

The change in water temperature also significantly affects the composition of the intestinal microbiota of fish. Pseudomonadota, Bacillota, Fusobacteriota, and Bacteroidota dominate the gut microflora of most marine and freshwater fish^48–50^. Our study indicated that in the gut microbiome of *L. longirostris*, the dominant flora of different temperature treatments were relatively consistent, and included Proteobacteria, Firmicutes, and Fusobacteriota. These three phyla have also been found to be dominant in the intestinal tracts of many other marine and freshwater fish specie^48,51^. In this study, although the different temperature treatments did not change the dominant bacteria in *L. longirostris*, they affected their relative abundances. The abundances of Pseudomonadota and Bacillota increased significantly in the cold stress group, while the abundances of Fusobacteriota and Bacteroidota increased significantly in the heat stress group. It has been reported that an increase in the abundance of Pseudomonadota in the host intestine may be caused by pathogenic bacteria^52^. *Aeromonas*, *Acinetobacter,* and *Cetobacterium* are Pseudomonadota, whereas *Aeromonas* and *Acinetobacter* are Gamma-pseudomonadota. Widely distributed in nature, these are typical opportunistic pathogens, which may cause many diseases such as enteritis and septicemia in humans and animals^53,54^. Bacillota produces short-chain fatty acids in the gut, and a higher abundance of Bacillota helps to obtain more energy from the diet^55^. Most of the bacteria in Fusobacteriota and Bacteroidota are typical anaerobic bacteria. In general, anaerobic bacteria and the bioprotective film formed by intestinal mucosa are the first barrier against pathogenic bacteria^56^. We speculate that the relative abundance of opportunistic pathogens in the intestinal tract of *L. longirostris* in the cold stress group was higher, and the resistance to diseases was relatively weaker when the internal environment was disturbed by environmental stress.

Alpha and beta diversity are important parameters for evaluating the level of organization of the community structure^57^. The higher the Chao1 index, the richer the community, and the larger the Shannon index, the greater the uniformity of the community. The species richness values of the temperature treatment groups were significantly lower than that of the control group, but the evenness values were slightly higher than that of the control group, indicating that the temperature treatments affected the richness and evenness of the intestinal microbiota. Beta diversity is an algorithm that converts the evolutionary relationship and abundance information of each sample sequence into an inter-sample distance, which directly reflects the community differences between the sample groups^57^. In this study, the two treatment groups and the control group were divided into three subgroups with statistical differences. Intestinal microbial diversity is closely related to the health of the body, and the decrease of intestinal microbial diversity may induce many diseases^58^. In addition, evidence shows that the functional composition of the microbial community is closely related to environmental factors, and different environments affect differences in microbial community functions^59,60^. Bacterial interaction analysis also found that Fusobacteriota and Proteobacteria were associated with most bacteria, which was consistent with the results of previous studies^61^. The functional prediction results showed that the functional abundance of the intestinal microbiota decreased significantly under the different temperature stressors. The precise causes of this phenomenon require further examination through analysis of the transcriptome and metabolome. In general, the intestinal microbial diversity of *L. longirostris* decreased, the abundance of pathogenic bacteria was higher, and intestinal functioning decreased in the cold- and heat-stress environments, particularly in the cold stress environment. Therefore, the *L. longirostris* intestinal microbiota detected in high or low temperature environment is not conducive to growth and resistance to disease, and the appropriate environmental temperature provides a better living environment for aquaculture animals.

## Conclusion

In this study, gill tissue sections and 16S rRNA sequencing technology were used to investigate the effects of different temperatures on the gill tissue structure and intestinal microbial homeostasis of *L. longirostris*. The cold- and heat-stress environments caused damage, swelling, terminal congestion, cellular vacuolation, and necrosis in the gills of *L. longirostris*. The stress causing ambient temperatures changed the composition of the intestinal microbiota, significantly affected the intestinal microbiota richness, and significantly reduced intestinal microbial function in *L. longirostris*. Based on these results, a suitable ambient temperature can maintain a rich gut microbiota, which has a stronger ability to resist disease for *L. longirostris*.

## Data Availability

The raw sequence data reported in this paper have been deposited in the Genome Sequence Archive^62^ in National Genomics Data Center^63^, China National Center for Bioinformation/Beijing Institute of Genomics, Chinese Academy of Sciences (GSA: CRA011773) that are publicly accessible at https://ngdc.cncb.ac.cn/gsa.
